# ATO (Arsenic Trioxide) Effects on Promyelocytic Leukemia Nuclear Bodies Reveals Antiviral Intervention Capacity

**DOI:** 10.1002/advs.201902130

**Published:** 2020-02-27

**Authors:** Samuel Hofmann, Julia Mai, Sawinee Masser, Peter Groitl, Alexander Herrmann, Thomas Sternsdorf, Ruth Brack‐Werner, Sabrina Schreiner

**Affiliations:** ^1^ Institute of Virology School of Medicine Technical University of Munich 85764 Munich Germany; ^2^ Institute of Virology Helmholtz Zentrum München 85764 Munich Germany; ^3^ Research Institute Children's Cancer Center Hamburg 20251 Hamburg Germany

**Keywords:** antivirals, arsenic, human adenoviruses, promyelocytic leukemia nuclear bodies, small ubiquitin‐like modifiers (SUMO)

## Abstract

Human adenoviruses (HAdV) are associated with clinical symptoms such as gastroenteritis, keratoconjunctivitis, pneumonia, hepatitis, and encephalitis. In the absence of protective immunity, as in allogeneic bone marrow transplant patients, HAdV infections can become lethal. Alarmingly, various outbreaks of highly pathogenic, pneumotropic HAdV types have been recently reported, causing severe and lethal respiratory diseases. Effective drugs for treatment of HAdV infections are still lacking. The repurposing of drugs approved for other indications is a valuable alternative for the development of new antiviral therapies and is less risky and costly than de novo development. Arsenic trioxide (ATO) is approved for treatment of acute promyelocytic leukemia. Here, it is shown that ATO is a potent inhibitor of HAdV. ATO treatment blocks virus expression and replication by reducing the number and integrity of promyelocytic leukemia (PML) nuclear bodies, important subnuclear structures for HAdV replication. Modification of HAdV proteins with small ubiquitin‐like modifiers (SUMO) is also key to HAdV replication. ATO reduces levels of viral SUMO‐E2A protein, while increasing SUMO‐PML, suggesting that ATO interferes with SUMOylation of proteins crucial for HAdV replication. It is concluded that ATO targets cellular processes key to HAdV replication and is relevant for the development of antiviral intervention strategies.

## Graphical abstract

Arsenic trioxide (ATO) treatment significantly represses efficient human adenovirus (HAdV) infection at low micromolar concentrations, involving reconstitution of dot‐like promyelocytic leukemia nuclear body structures, which thereby maintain their antiviral function. Intervention of dsDNA virus gene expression and replication by improving the host antiviral defense mechanisms provide a novel basis for innovative antiviral strategies in future therapeutic settings.

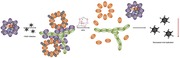

## 1 Introduction

Human adenoviruses (HAdV) are large, non‐enveloped icosahedral viruses containing a double stranded (ds) DNA genome. Since their isolation, HAdV have been extensively studied and were used as a model system to provide insights into many biological pathways of human cells. For instance, the discovery of alternative mRNA splicing or the investigation of apoptosis, cell cycle control, and tumorigenesis, are based on HAdV research.^1^, ^2^, ^3^, ^4^ Additionally, HAdV are subject to extensive research as they can be used as therapeutic gene transfer and vaccine vectors. In fact, HAdV are among the most commonly used viruses as gene transfer vectors in clinical trials.^5^

Currently, there are 89 HAdV types identified,^6^ which are divided into seven species A–G. The different groups of HAdV are additionally characterized by different tissue tropism, which determine the site of primary infection. HAdV mostly infect the respiratory tract, causing pharyngitis or pneumonia (B1, C, E), the gastrointestinal tract (A, F), the renal system (B2), or are the cause of ocular diseases as, for example, viral keratokonjunctivitis (D).^7^ HAdV contribute to about 8% of respiratory tract infections in infants. However, HAdV infections are mostly subclinical and self‐limiting in immunocompetent individuals.^8^ However, in immuncompromised populations, like solid‐organ transplant or hematopoietic stem cell transplant recipients and patients suffering from cancer or additional chronic infections, HAdV infections can lead to severe complications with high morbidity and mortality.^9^, ^10^ Despite being subject to extensive research and their high clinical impact, neither specific anti‐adenoviral treatment nor an efficient vaccination strategy are available yet.^11^

Promyelocytic leukemia nuclear bodies (PML‐NBs) or PML oncogenic domains (POD) are nuclear dot‐like multi‐protein complexes, which are associated with the nuclear matrix.^12^, ^13^, ^14^ These interferon‐inducible nuclear structures are important regulators, participating in various cellular processes like senescence, apoptosis, transcription and epigenetic regulation, protein degradation, and antiviral defense.^15^, ^16^, ^17^, ^18^, ^19^ Besides PML, other constitutively expressed proteins, such as Sp100, Daxx, and small ubiquitin‐like modifier (SUMO) are found in the PML‐NBs.^20^ PML‐NBs are important modulators of viral infection, as several interferon‐induced components, such as PML, Daxx, or Sp100 can impair efficient viral replication.^15^, ^21^, ^22^, ^23^ To counteract these measures, several DNA viruses express early viral proteins, which interact with PML‐NBs and specifically interfere with their antiviral function. On the other hand, viral genomes and replication centers are often found juxtaposed to PML‐NBs in the nucleus of the host cell, indicating a positive influence of some PML components on viral replication.^24^

In cells infected with HAdV, PML‐NBs are reorganized from the usual dot‐like into so called track‐like structures by oligomerization of E4orf3.^25^ This disrupts the integrity of PML‐NBs and inhibits the cellular antiviral interferon response by sequestration of Sp100 and Daxx.^26^, ^27^, ^28^ Subsequently in the course of infection, the function of several PML‐NB associated proteins with anti‐adenoviral activity including p53, Daxx, and the MRN complex are disrupted by early HAdV proteins, a step necessary for efficient HAdV replication.^29^, ^30^, ^31^, ^32^, ^33^, ^34^, ^35^, ^36^, ^37^, ^38^, ^39^, ^40^, ^41^ Additionally, it was shown that adenoviral DNA replication takes place at sites juxtaposed to PML‐NBs, and that the PML‐NB components Sp100A and Usp7, but not PML itself, are recruited to the replication centers.^42^, ^43^, ^44^ Recently, we provided evidence that targeting of the viral replication centers to sites of PML‐NBs before track formation is not dependent on interactions of PML‐NB components and the incoming viral genome, but is mediated by SUMOylation of the viral E2A protein promoting PML and Sp100A interaction with the viral DNA binding factor.^45^

ATO (arsenic trioxide/As_2_O_3_) is a compound used for over 2000 years in traditional Chinese medicine. In 1992, it was first described to induce an efficient and complete response in patients suffering from acute promyelocytic leukemia (APL)^46^, ^47^, ^48^ prior to approval by the U.S. Food and Drug Administration (FDA) for APL treatment in 2000.^49^ In APL disease, PML (the main component of PML‐NBs) is fused to the retinoic acid receptor alpha (RARA) due to a chromosomal translocation. This genetic disorder induces an aberrant track‐like structure of PML‐NBs resulting in a complete disruption of their functionality.^50^, ^51^, ^52^ Treatment of cells with ATO leads to a direct binding of arsenic to the PML protein, mainly via interaction with cysteine residues within PML.^53^, ^54^ It thereby induces oxidation of PML, prior to PML multimerization at early time points, reconstituting the normal dot‐like structure of PML‐NBs in APL patient cells.^53^ At later time points, PML and PML‐NB components undergo hyper‐SUMOylation, which is subsequently followed by ubiquitinylation of those proteins by the SUMO‐dependent E3‐ubiquitin ligase RNF4, leading to proteasomal degradation.^53^, ^54^, ^55^, ^56^

In summary, disruption of PML‐NB integrity is essential for efficient HAdV replication and progeny production. Here, we speculated that HAdV mediated relocalization of PML‐NBs into tracks seems at a first glance similar to the PML‐NB microspeckled phenotype in APL cancer cells. Thus, we hypothesized, that ATO treatment would negatively regulate efficient HAdV infection cycle, involving oxidation of PML, PML multimerization and PML hyper‐SUMOylation to reconstitute the normal dot‐like structure of PML‐NBs. In our study, we observed that ATO showed significant in vitro anti‐HAdV activity at low micromolar concentrations with reduced cytotoxicity. ATO acted as a potent inhibitor of HAdV infection. Inhibition was likely due to alteration of SUMOylation processes and the reconstitution of the typical dot‐like shape of PML‐NBs, which thereby maintain their antiviral function. Thus, our study reveals a novel and general impact of ATO treatment on intervention of dsDNA virus gene expression and replication by improving the host antiviral defense mechanisms, providing a novel basis for innovative antiviral strategies in future therapeutic settings.

## 2 Results

### 2.1 ATO Antagonizes Productive HAdV Infection Cycles in Human Lung Cells

**Figure 1:**
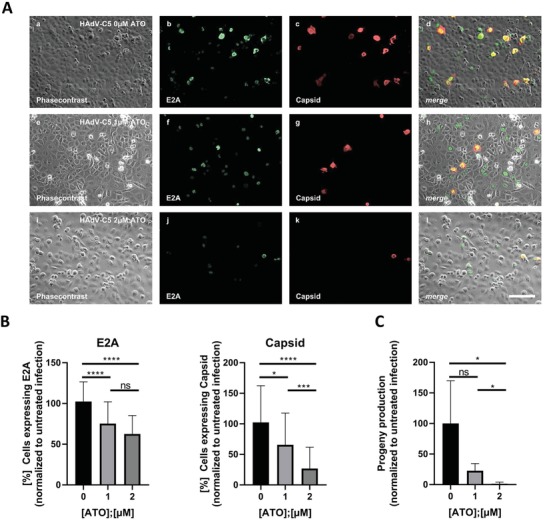
ATO efficiently reduces HAdV progeny production. A,B) H1299 cells were infected with HAdV‐C5 wt at a multiplicity of 5 FFU per cell, treated with the depicted concentrations of ATO at 2 h p.i., fixed 48 h p.i. with 4% PFA and double labeled with mAb B6‐8 (α‐E2A) and pAb L133 (α‐capsid). Primary antibodies were detected using Alexa488 (E2A, green) and Alexa568 (capsid, red) conjugated secondary antibodies. A) Representative overview pictures of *n* = 40 pictures are shown. Scale bar represents 200 µm. B) The number of E2A and capsid expressing cells, respectively, was counted in *n* = 40 overview pictures, normalized to untreated, infected cells, and represented in bar charts. Bar charts represent average values and standard deviations based on four independent experiments. C) H1299 cells were infected with HAdV‐C5 at a multiplicity of 20 FFU per cell, and treated with the depicted concentrations of ATO at 2 h p.i. Viral particles were harvested 48 h p.i. and virus yield was determined by quantitative E2A immunofluorescence staining in 293 cells. Bar charts represent average values and standard deviations based on three independent experiments. Statistically significant differences were determined using a one‐way ANOVA and Dunnet's T3 test. *p ≤ 0.05, **p ≤ 0.01, ***p ≤ 0.001, ****p ≤ 0.0001.

Previous studies on ATO effects in cell culture used concentrations lower than 2.0 µm.^55^, ^57^, ^58^ Clinical studies on pharmacokinetics showed that the arsenic serum concentrations, and therefore the therapeutic range upon oral and i.v. administration in APL patient treatment lies between 1.0 and 2.0 µm to peak levels of 7.3 µm.^46^, ^59^ Thus, we used ATO concentrations between 0 and 2.0 µm. Here, we first infected human lung carcinoma cells with HAdV wt virus and treated with different concentrations of ATO. These initial studies determined a dose‐dependent anti‐HAdV activity with 40% reduction of early viral DNA binding protein E2A expression at 1 µm, and almost 80% inhibition of late capsid protein expression at 2.0 µm ATO (**Figure** **1**A,B).

Next, we evaluated whether ATO was also able to inhibit virus progeny production. We observed that treatment with 1 µm ATO was associated with 80% reduction of virus yield, while 2 µm ATO abolished virus particle synthesis compared to non‐treated and infected control cells (Figure 1C).

### 2.2 ATO Blocks Efficient HAdV Gene Expression

**Figure 2:**
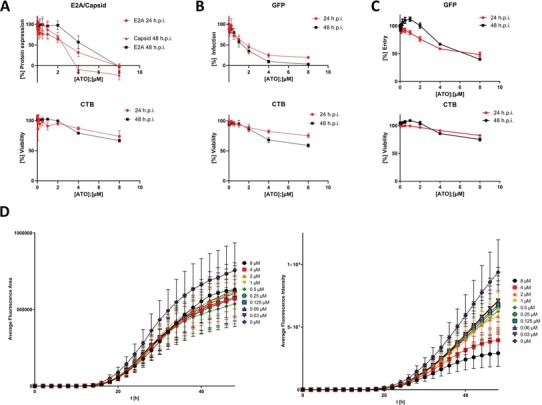
ATO induces a dose dependent reduction of HAdV infectivity with minor effects on cell viability. A) H1299 cells were infected with HAdV‐C5 wt at a multiplicity of 20 FFU per cell and treated with the depicted concentrations of ATO at 2 h p.i. 24 or 48 h p.i., cell viability was assessed using the Promega CellTiter‐Blue Cell Viability Assay system, prior to fixation with 4% PFA and cells were double labeled with mAb B6‐8 (α‐E2A) and pAb L133 (α‐capsid). Primary antibodies were detected using Alexa488 (E2A, green) and Alexa647 (capsid, red) conjugated secondary antibodies. Fluorescence intensity was measured using a Tecan Infinite 200M plate reader using an excitation and emission wavelength of 488 and 520 nm for Alexa488 and 640 and 670 nm for Alexa647, respectively. Fluorescence intensity was normalized to untreated, infected cells. *xy* charts represent average values and standard deviations based on three independent experiments measured in triplicates. B) H1299 cells were infected with an HAdV‐C5 delta E3 virus, containing a CMV promoter driven eGFP expression cassette, at a multiplicity of 20 FFU per cell and treated with the depicted concentrations of ATO at 2 h p.i. 24 or 48 h p.i. cell viability was assessed using the Promega CellTiter‐Blue Cell Viability Assay system, and GFP fluorescence intensity was measured using a Tecan Infinite 200M plate reader using an excitation and emission wavelength of 488 and 520 nm. Fluorescence intensity was normalized to untreated, infected cells. *xy* charts represent average values and standard deviations based on three independent experiments measured in triplicates. C) H1299 cells were infected with an eGFP expressing HAdV‐C5‐based first generation adenoviral vector at a multiplicity of 20 FFU per cell and treated with the depicted concentrations of ATO at 2 h p.i. 24 or 48 h p.i., cell viability was assessed using the Promega CellTiter‐Blue Cell Viability Assay system, and GFP fluorescence intensity was measured using a Tecan Infinite 200M plate reader using an excitation and emission wavelength of 488 and 520 nm. Fluorescence intensity was normalized to untreated, infected cells. *xy* charts represent average values and standard deviations based on three independent experiments measured in triplicates. D) A549 cells were infected with an HAdV‐C5 delta E3 virus, encoding a CMV promoter driven eGFP expression cassette, at a multiplicity of 20 FFU per cell and treated with the depicted concentrations of ATO at 2 h p.i. GFP‐fluorescence, as well as cell growth via phase‐contrast imaging was assessed for 48 h with a 2 h increment using an IncuCyte S3 Live‐Cell Analysis System. Average fluorescence area (left) and average fluorescence intensity (right) were determined and plotted over the time.

We then monitored the effect of treatment with different ATO concentrations (0–8 µm) on levels of the E2A protein (24/48 h post infection [p.i.])/capsid (48 h p.i.). Treatment with 4 µm ATO reduced E2A levels at 24 h p.i. to less than 50% of untreated controls and treatment with 8 µm ATO completely blocked HAdV E2A protein expression (IC50 1.910 µm; **Figure** **2**A). At 48 h p.i., 4 µm ATO reduced E2A expression to 60% and E2A was not detectable with 8 µm ATO (IC50 4.062 µm). For capsid expression at 48 h p.i., we observed a 40% reduction with 2 µm ATO and complete loss of capsid detection with 4 µm ATO (IC50: 2.067 µm). The cellular cytotoxicity of ATO in these assays were analyzed simultaneously and showed no strong effect (Figure 2A, lower panel). Using HAdV wt infection, we showed that ATO efficiently reduces the number of cells infected with adenovirus and expressing early E2A and late capsid protein (Figure 2A).

To further validate significant reduction of HAdV gene expression, we generated a replication competent HAdV containing a GFP expression cassette under the transcriptional control of the CMV promoter. Quantification of GFP served as a measure for productive HAdV infection. Here, we observed highly efficient inhibition of HAdV infection with ATO after 24 and 48 h p.i. (Figure 2B). Our data indicate 50% reduction of HAdV infection under treatment with 2 µm ATO after 24 h p.i. (IC50 1.610 µm), which was even stronger after 48 h p.i. (IC50 1.177 µm). Again, the cellular cytotoxicity of ATO in these assays was analyzed simultaneously and showed no strong effect (Figure 2B, lower panel).

To clarify ATO capacity to block viral gene expression in the absence of viral spread, we carried out an assay to measure viral transcript levels, using a non‐replicating eGFP expressing HAdV‐C5‐based first generation adenoviral vector. Here, we applied different ATO concentrations (0–8 µm) and measured GFP protein concentrations. Less than 50% GFP expression compared to the untreated control was only observed with 8 µm ATO after 24 h (IC50 6.485 µm) and 48 h p.i. (IC50 10.240 µm; Figure 2C). The cellular cytotoxicity of ATO in these assays was analyzed simultaneously and showed no strong effect (Figure 2C, lower panel).

To further validate inhibition of HAdV by ATO, we determined real‐time kinetic data with a dilution of different ATO concentrations after HAdV‐GFP wt infection and monitored every second hour up to 48 h p.i. with an IncuCyte automated live‐cell imaging system. Here, fluorescence images were taken every 2 h for 48 h in a 37 °C CO_2_ incubator. We substantiated our data presented above, and also observed that ATO efficiently repressed GFP expression over time with a fourfold reduction at 48 h p.i. (Figure 2E).

### 2.3 ATO Reduces HAdV DNA and RNA Synthesis

**Figure 3:**
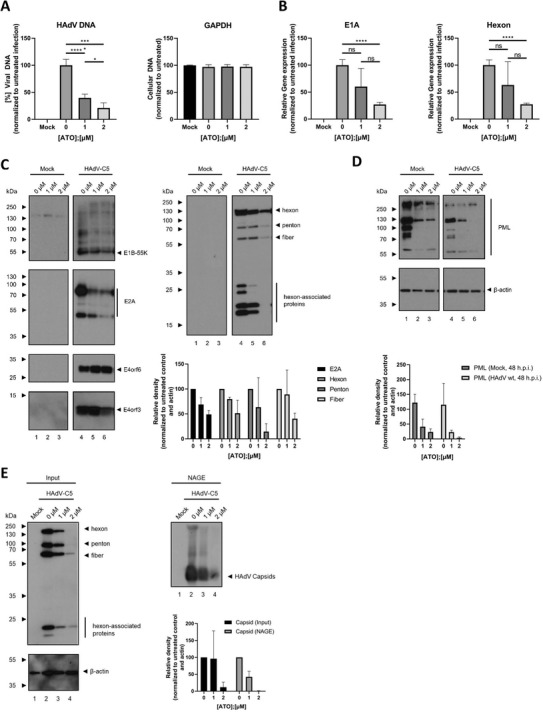
ATO interferes with HAdV gene expression and capsid formation. H1299 cells were infected with HAdV‐C5 wt at a multiplicity of 20 FFU per cell and treated with the depicted concentrations of ATO at 2 h p.i. A) Cells were harvested 24 h p.i., total DNA was isolated and subjected to qPCR using primers specific for the viral *e1b* coding region. As internal control, primers for GAPDH were used. Bar charts represent average values and standard deviations based on two independent experiments measured in triplicates. B) Cells were harvested 48 h p.i. and total mRNA was isolated using TRIzol, reverse transcribed and quantified by RT‐PCR using primers specific for HAdV E1A and hexon. The data was normalized to the respective 18S mRNA levels. Bar charts represent average values and standard deviations based on two independent experiments measured in triplicates. C) 48 h p.i., proteins from total‐cell protein lysates were separated by SDS‐PAGE and subjected to immunoblotting using mAb 2A‐6 (α‐E1B‐55K), mAb RSA3 (α‐E4orf6), mAb B6‐8 (α‐E2A), mAb6A11 (α‐E4orf3), and pAb L133 (α‐capsid). Relevant proteins are depicted on the right, molecular weights in kDa on the left of each blot, respectively. For quantification of protein expression, densitometric analysis of detected bands was performed using ImageJ (version 1.45s). Relative protein expression was normalized on the respective α‐β‐actin steady state levels. Bar charts represent average values and standard deviations based on three independent experiments. D) Protein lysates from C were separated by SDS‐PAGE and subjected to immunoblotting using pAb NB100‐59787 (α‐PML), and mAb AC‐15 (α‐β‐actin). Relevant proteins are depicted on the right, molecular weights in kDa on the left of each blot, respectively. For quantification of protein expression, densitometric analysis of detected bands was performed using ImageJ (version 1.45s). Relative protein expression was normalized on the respective α‐β‐actin steady‐state levels. Bar charts represent average values and standard deviations based on three independent experiments. E) Cells were lysed using a low stringent NP‐40 lysis buffer. Native lysates were separated by agarose gel electrophoresis and further analyzed by immunoblotting using pAb L133 (α‐capsid). For the determination of protein steady‐state levels, lysates were denatured using Laemmli buffer, separated by SDS‐PAGE, and subjected to immunoblotting using pAb L133 (α‐capsid) and mAb AC‐15 (α‐β‐actin). Relevant proteins are depicted on the right, molecular weights in kDa on the left of each blot, respectively. For quantification of protein expression, densitometric analysis of detected bands was performed using ImageJ (version 1.45s). Relative protein expression was normalized on the respective α‐β‐actin steady‐state levels. Relative capsid levels as detected by NAGE were normalized to input capsid expression levels, as well as the respective α‐β‐actin steady‐state levels. Bar charts represent average values and standard deviations based on three independent experiments. Statistically significant differences were determined using a one‐way ANOVA and Dunnet's T3 test. *p ≤ 0.05, **p ≤ 0.01, ***p ≤ 0.001, ****p ≤ 0.0001.

Next, we examined the effect of ATO upon efficient HAdV DNA replication using quantitative real‐time PCR (qPCR). Cells were infected with HAdV and incubated for 24 h or 48 h. HAdV DNA was extracted and quantitative PCR was performed to quantify the synthesis of new HAdV DNA as a measure of DNA replication efficiency. ATO treatment significantly inhibited HAdV DNA replication at 1 µm (60% reduction) and 2 µm (80% reduction) of the drug (**Figure** **3**A). No effect on levels of GAPDH DNA was observed, which served as control.

To assay the inhibition of HAdV mRNA transcription by ATO, we infected cells in the presence of the compound (0–2 µm) for 2 h p.i. and harvested 48 h later. After infection, we quantified the mRNA copy number of E1A (early gene) and Hexon (late gene) using quantitative RT‐PCR. ATO treatment reduced total E1A/Hexon mRNA levels (Figure 3B), revealing 80% (E1A) and 60% (Hexon) inhibition of viral mRNA expression at 2 µm, without significant impact of the compound on host 18S mRNA. Thus, ATO directly affects early and late viral mRNA production.

### 2.4 ATO Significantly Reduces HAdV Protein Synthesis

Western blot analysis of viral protein expression substantiated the above findings, revealing significantly reduced viral early and late protein synthesis under 1 and 2 µm ATO treatment (Figure 3C). Consistent with the delay in viral mRNA synthesis under ATO treatment, we also detected a decrease in expression of the early viral protein E2A and a decrease in late capsid and capsid associated proteins (Figure 3C). Under ATO treatment, we detected a decrease in E1B‐55K and a minor reduction of E4orf6 viral proteins (Figure 3C). These two viral factors are viral components of an E3 ubiquitin ligase complex, which is essential for efficient virus replication due to proteasomal degradation of host substrates. In line with these facts, we observed insufficient proteasomal degradation of these substrates in infected cells treated with ATO compared to untreated cells (data not shown).

### 2.5 ATO Induces Modulation of PML and PML‐Associated E4orf3 Protein Levels during HAdV Infection

In APL disease, PML‐NBs are disrupted in the cell nucleus with a consequent loss of their tumor‐suppressive activity. ATO induces the subsequent reformation of PML‐containing NBs by SUMOylation of the PML/PML‐RAR alpha protein, concomitant recruitment of a SUMO‐dependent ubiquitin ligase (RNF4), and polyubiquitinylation. Finally, PML and RAR alpha proteins are degraded by the proteasome.^60^, ^61^ We previously reported that during HAdV productive infection, RNF4 is recruited into the insoluble matrix^33^ and PML‐NB integrity is disrupted by relocalization into track‐like structures.^62^, ^63^ Here, we monitored PML expression during HAdV infection under ATO treatment. ATO mediated reduction of PML was observed independent of HAdV infection (Figure 3D). We also observed that viral E4orf3 protein expression was reduced by 2 µm ATO treatment (Figure 3C, left panel). E4orf3 promotes the reorganization of PML‐NBs by interacting with PML‐II isoform, which leads to the formation of track‐like structures in the nucleus of infected cells.^62^, ^63^, ^64^, ^65^, ^66^, ^67^, ^68^ This reorganization of PML‐NBs is highly conserved among most HAdV species^69^; and therefore suggests an important function during HAdV infection, presumably in eliminating intracellular viral defense barriers.^15^, ^23^, ^24^, ^62^

### 2.6 ATO Interferes with Efficient Virus Particle Assembly

Next, we monitored the impact of ATO treatment on HAdV assembly. Thus, we performed a low stringent lysis of infected cells in the presence of ATO prior to separation of native lysates with agarose gel electrophoresis and immunoblotting using capsid antibody. We detected capsid formation without ATO treatment in infected cells (Figure 3E, right panel). Our native gel assay showed that ATO presence significantly reduced the signal obtained after capsid staining. However, capsid protein expression was also reduced (Figure 3E, right panel). Normalization to capsid input (Figure 3E) strengthened the observation that ATO reduced HAdV capsid assembly (Figure 3E, right panel).

### 2.7 ATO Treatment Affects the Number and Structure of PML Bodies and Suppresses Formation of HAdV Replication Centers

**Figure 4:**
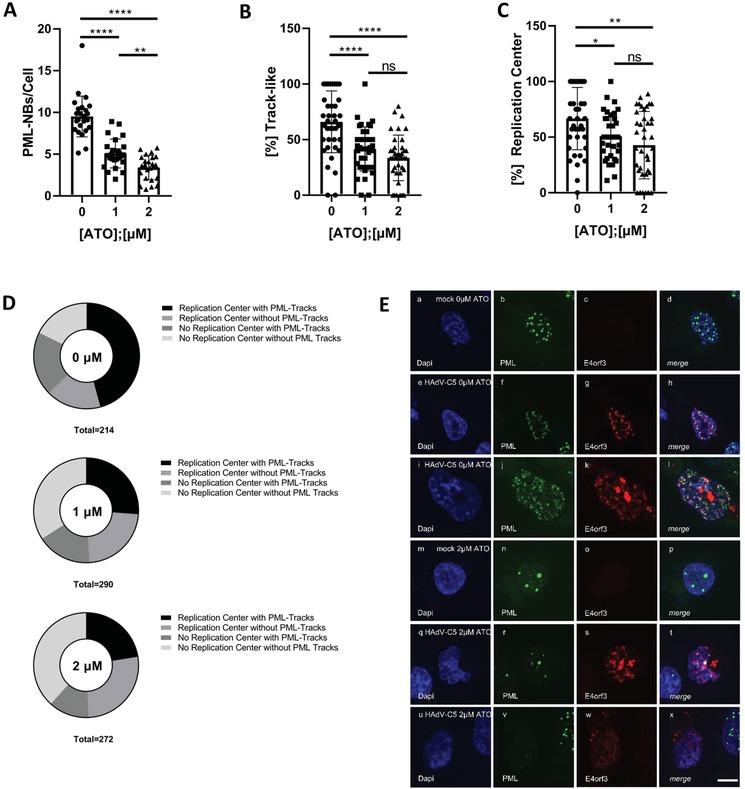
ATO interferes with efficient HAdV replication center formation and reorganization of PML‐NBs. H1299 cells were infected with HAdV‐C5 wt at a multiplicity of 20 FFU per cell, treated with the depicted concentrations of ATO at 2 h p.i., fixed 48 h p.i. with 4% PFA and double labeled with mAb B6‐8 (α‐E2A) and pAb NB100‐59787 (α‐PML). Primary antibodies were detected using Alexa488 (PML, green) and Alexa647 (E2A, red) conjugated secondary antibodies. A) Number of PML‐NBs per cell in uninfected cells was determined using Volocity for at least *n* = 461 cells from two independent biological replicates. Statistically significant differences were determined using a one‐way ANOVA and Dunnet's T3 test. *p ≤ 0.05, **p ≤ 0.01, ***p ≤ 0.001, ****p ≤ 0.0001. B) The proportion of infected cells showing track‐like redistribution of PML‐NBs was determined by counting for at least *n* = 212 cells and normalization to untreated infected cells. C) The proportion of infected cells showing formation of HAdV replication centers marked by the viral protein E2A (lower plot) was determined by counting for at least *n* = 212 cells and normalization to untreated infected cells. D) Cells showing either viral replication centers with PML track‐like structures, replication centers without PML track‐like structures, no replication centers but PML track‐like structures or no replication centers, and no PML track‐like structures were counted for at least *n* = 214 (virus infected cells due to E2A signal detected: either untreated/0 µm or treated with ATO/1 or 2 µm) and represented in pie charts. Statistically significant differences were determined using a one‐way ANOVA and Dunnet's T3 test. *p ≤ 0.05, **p ≤ 0.01, ***p ≤ 0.001, ****p ≤ 0.0001. E) H1299 cells were infected with HAdV‐C5 wt at a multiplicity of 20 FFU per cell, and treated with 0 or 2 µm of ATO at 2 h p.i. After 48 h, the cells were fixed with 4% PFA and stained using pAb NB100‐59787 (α‐PML) and mAb 6A‐11 (α‐E4orf3). Primary antibodies were detected using Alexa488 (PML, green) and Alexa647 (E4orf3, red) coupled secondary antibodies. Representative staining patterns for at least 30 uninfected cells treated with 0 or 2 µm ATO are shown in panels (a)–(d) and (m)–(p), infected cells treated with 0 or 2 µm ATO are shown in panels (e)–(l) and (q)–(x). Overlays of single fluorescence pictures (merge) are shown in panels (d), (h), (l), (p), (t), and (x). Data corresponds to two independent biological replicates performed and counted by different operators to avoid operator bias. Scale bar represents 10 µm.

To further analyze the ATO impact on the number of PML‐NBs and HAdV mediated PML tracks, we performed intracellular immunofluorescence studies in mock and infected cells (**Figure** **4**). Consistent with literature, ATO treatment significantly decreased the number of PML‐NBs in non‐infected cells (Figure 4A). Our data also indicate reduction of track‐like structures in infected cells when ATO is present (Figure 4B).

HAdV localize their genomes juxtaposed to PML‐NBs,^24^, ^42^, ^62^, ^70^, ^71^, ^72^ as formation of replication and transcription domains (RCs; replication centers) often takes place in close proximity to PML‐NBs.^73^ Intriguingly, after ATO treatment in HAdV infected cells, we observed a significant reduction of RC in number and a severe impact on the formation of RCs juxtaposed to virus‐induced PML track‐like structures (Figure 4C,D). In 60% of all infected cells without ATO treatment, we detected RCs and juxtaposed PML‐tracks (Figure 4D). All other combinations (RCs/no PML tracks; no RCs/PML tracks; no RCs/PML tracks) were equally distributed amongst all other infected cells (Figure 4D). Once ATO was present, cells with RCs and juxtaposed PML tracks were reduced to only 30% of the cells infected with HAdV (Figure 4D). ATO showed no significant impact on other identified combinations, such as RCs/no PML tracks, no RCs/PML tracks, no RCs/PML tracks.

Next, we monitored E4orf3 localization in ATO treated and HAdV infected cells (Figure 4E). This viral protein promotes reorganization of PML‐NBs into track‐like structures.^62^, ^63^, ^64^, ^65^, ^66^, ^67^, ^68^ Here, we observed that in untreated cells, E4orf3 colocalized with PML track structures induced by HAdV infection. However, in infected cell treated with 2 µm ATO, we still detected E4orf3 in track‐like structures. Intriguingly, PML did not localize to track‐like structures, and PML appeared in nuclear bodies that resemble those detected in mock‐treated cells. This indicates that E4orf3 containing track‐like structures are formed also in the absence of PML under ATO treatment.

### 2.8 ATO Interferes with E2A SUMOylation during HAdV Infection

**Figure 5:**
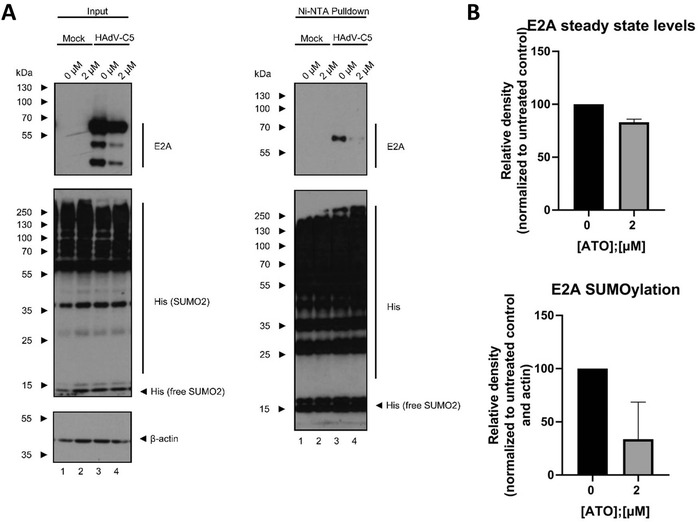
ATO highly reduces SUMO2 modification of E2A. A) H1299 cells were transfected with 10 µg of p6xHis‐SUMO2 for 4 h. After transfection, cells were infected with HAdV‐C5 wt at a multiplicity of 20 FFU per cell, treated with 2 µm of ATO and harvested 24 h p.i. Whole‐cell lysates were prepared with guanidinium chloride buffer and subjected to Ni‐NTA purification of 6His‐SUMO2 conjugates. After Ni‐NTA purification, the 6His‐SUMO2 conjugates, as well as proteins from total‐cell protein lysates were separated by SDS‐PAGE and subjected to immunoblotting using mAb B6‐8 (α‐E2A), mAb 6His (α‐6xHis‐tag), and mAb AC‐15 (α‐β‐actin). Relevant proteins are depicted on the right, molecular weights in kDa on the left of each blot, respectively. B) For quantification of protein expression, densitometric analysis of detected bands was performed using ImageJ (version 1.45s). Relative protein expression was normalized on the respective α‐β‐actin steady‐state levels. The degree of E2A SUMO2 modification was further normalized on E2A steady‐state levels. Bar charts represent average values and standard deviations based on three independent experiments.

Due to its essential role in HAdV replication, E2A is termed as marker for HAdV replication centers,^74^ located juxtaposed to PML tracks.^24^, ^42^, ^62^, ^70^, ^71^, ^72^ Furthermore, viral E2A was previously demonstrated to interact with PML.^45^ We reported earlier that the host SUMOylation machinery targets E2A, facilitating E2A protein functions and host interactions. E2A SUMOylation represents the molecular mechanism determining PML track subcellular localization adjacent to HAdV RCs.^45^ Thus, we next investigated E2A SUMOylation status in ATO treated infected cells transiently transfected with p6xHis‐SUMO2. We performed NiNTA pull down assays in infected cells under 2 µm ATO treatment (**Figure** **5**A). Our data showed that ATO significantly decreased the presence of E2A SUMO moieties in infected cells by 70% compared to untreated cells. These results correspond to the above made observations that ATO reduces the distance between RCs and juxtaposed PML tracks by direct modulation of E2A SUMOylation status.

## 3 Discussion

During the years, the incidence of severe HAdV infections has extensively increased. A high clinical impact is observed in both immunosuppressed and immunocompetent patients. In this context, the lack of a specific drug to treat these infections supports the search for new therapeutic alternatives. In this study, we examined the anti‐HAdV properties of ATO, a commercially available drug used for decades in APL disease.

HAdV manifestations are associated with severe clinical symptoms and often result in disseminated and potentially life‐threatening disease with growing numbers of fatalities in immune‐compromised patients, such as in AIDS patients, in individuals with hereditary immunodeficiencies and recipients of solid organ or hematopoietic stem cell transplants (HSCT). Multiple studies have determined a mortality rate of 4–6%, most presumably due to a fatal cytokine storm.^75^ Severe organ infections or even disseminated disease with high virus loads in peripheral blood, multi‐organ failure, and sepsis‐like symptoms show a lethality rate of up to 70%. Manifestations can arise from de novo infections but probably more frequently from latent HAdV infections.^76^, ^77^ However, until today, there is no effective chemotherapeutic drug available to treat HAdV infections. Commonly used antiviral treatment, which was mainly based on nucleoside or nucleotide analogues, such as cidovovir and ribavirin, only limit HAdV but failed to cure severe infection and rescue the patient.^78^, ^79^, ^80^, ^81^, ^82^, ^83^

ATO is very potent as a single agent against acute promyelocytic leukemia. This ancient drug was reintroduced into modern medicine by Chinese studies on APL treatment and rapidly approved by the FDA for relapsed cases. Recently, ATO has undergone studies for other hematologic and non‐hematologic malignancies and will probably be useful in future, in combination with other drugs in these disorders.^84^

ATO binds to the PML component of PML‐RARA through two cysteine residues in the B2 domain of PML protein, inducing their oxidation, leading to disulfide bond formation. The subsequent reformation of PML containing nuclear bodies allows SUMOylation of the PML/PML‐RARA protein, concomitant recruitment of a SUMO‐dependent ubiquitin ligase (RNF4) and polyubiquitinylation. The final step is the degradation of the PML moiety and its associated RARA partner by the proteasome. Additionally, ATO induces the degradation of PML/PML‐RARA through the production of reactive oxygen species (ROS) that also produces oxidation of PML and formation of nuclear bodies.^53^, ^54^, ^55^, ^56^

A potential use for ATO as antiviral compound was first proposed by Kuroki et al., who suggested an inhibitory effect of ATO on hepatitis C virus replication by the induction of reactive oxygen species, independent of its effect on PML.^85^ Here, we report a different mode of action in the inhibition of HAdV replication, relying on the impact of ATO on PML.

Here, we show that ATO treatment efficiently reduced HAdV early and late protein expression with IC50 values in the low micromolar range, similar to the observed serum concentrations of 1.0–2.0 µm in APL patients.^46^, ^59^ Additionally, treatment of infected cells with 2 µm ATO significantly diminished viral progeny production, indicating that ATO is a highly potent drug to treat severe and life‐threatening HAdV infections.

To establish infection, HAdV early proteins impair PML‐NB integrity, achieved by E4orf3 oligomerization and interaction with PML‐II isoform.^62^ Within these disrupted PML bodies, E1A‐13S was shown to target the PML‐II isoform to efficiently activate viral and virus‐promoting host transcription.^86^ Western blot analysis of ATO treated, HAdV infected cells showed a marked delay in the expression of the HAdV immediate early E1A protein, suggesting that the activation and enhancement of HAdV gene expression by interaction of E1A and PML‐II is impaired due to rearrangement and oxidative hyper‐SUMOylation of PML by ATO treatment.^53^, ^54^, ^55^, ^56^ This finding is consistent with a decrease in the subsequently expressed other early viral proteins, like E2A, and a decrease in viral DNA synthesis. We speculate, that the more pronounced reduction in late viral protein expression might be a secondary effect, evoked from the reduction in early viral protein production and also viral DNA synthesis, as generation of new viral genomes is essential for late HAdV protein synthesis.^87^, ^88^ This deficiency in viral DNA synthesis might also contribute to the reduction in viral particle formation observed in the native agarose gelectrophoresis.

During HAdV infection, PML‐NBs are relocalized from their typical dot‐like structure into a filamentous network, so‐called track‐like structures, induced by E4orf3 oligomerization and interaction with PML‐II isoform.^62^ Using this, among different HAdV species highly conserved mechanism, host factors beneficial for efficient virus infection are recruited into PML tracks. Simultaneously, antiviral proteins, such as p53, Mre11, ATRX, SPOC1, etc. are removed from the infected cell via the host proteasomal pathways and the viral E1B‐55K/E4orf6‐based E3‐ubiqutin ligase complex.^86^, ^89^, ^90^, ^91^, ^92^ ATO treatment hereby strongly impaired the ability of HAdV to reorganize PML‐NBs into E4orf3 containing track‐like structures, and therefore inhibit the antiviral activity. Additionally, the block of PML track formation by ATO treatment reduced the functionality of the viral E1B‐55K/E4orf6‐based ubiquitin ligase complex to contribute to the degradation of cellular components. This could either be a direct effect of the reduction in formation of PML track‐like structures, which might prevent access of the viral E3‐ubiquitin ligase to their substrates, or a direct effect of ATO on post‐translational modifications of HAdV proteins including SUMOylation.

As intracellular pathogens and cellular stressors, HAdV are exposed to, and also induce PTMs during multiple stages of infection.^93^ In detail, modulation of the SUMOylation machinery during virus infection plays an important role and is needed to promote efficient replication.^94^, ^95^ During HAdV‐C5 infections, the involvement of the SUMO proteins has been studied extensively for the expressed proteins E1A, E1B‐55K, E2A, E4orf3, and pV.^45^, ^96^, ^97^ Here, the HAdV E4orf3 protein induces SUMOylation of multiple cellular proteins, such as the DDR components, Mre11 and Nbs1, which are then targeted for E1B‐55K/E4orf6‐mediated proteasomal degradation upon transient SUMOylation.^98^ Furthermore, SUMOylation of the cellular transcription factors TFII‐I and TIF‐1γ is increased in the presence of E4orf3 and are degraded through an E4orf3‐dependent, E1B‐55K/E4orf6‐independent pathway.^99^, ^100^ Moreover, E4orf3 functions as a SUMO E3 ligase and a SUMO E4 elongase toward its target proteins.^101^, ^102^ Vice versa, SUMO modification is essential for the assembly of the intranuclear structures PML‐NBs. With ATO inducing oxidative hyper‐SUMOylation of PML,^53^, ^54^, ^55^, ^56^ an interference of ATO with post‐translational modification of HAdV proteins with SUMO, for example, by depletion of the SUMO pool might be possible. Indeed, the NiNTA pulldown in cells transfected with His_6_‐tagged SUMO2 showed that SUMO2 modification of the viral DNA binding protein E2A was strongly reduced upon treatment with ATO. As PTM of E2A with SUMO2 is a prerequisite for the juxtaposition of HAdV replication centers to PML track‐like structures, in order to promote viral replication,^45^ the reduction in SUMOylation of E2A supposedly contributes to the observed decrease in replication center formation and also HAdV replication in general. In addition, recruitment of E1B‐55K to PML‐NBs, as well as the E3‐SUMO ligase function of the viral protein depends on E1B‐55K SUMOylation.^34^, ^35^, ^91^, ^103^, ^104^ A possible depletion of the cellular SUMO pool by ATO might therefore contribute to a deficiency in SUMO modification of E1B‐55K, and thereby impair its essential function during the viral life cycle.

**Figure 6:**
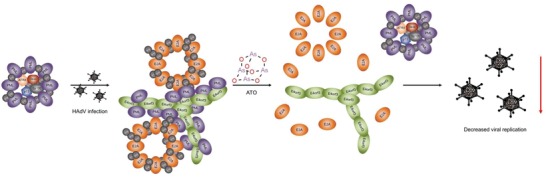
Proposed mode of action of ATO during HAdV infection. Based on our data, we hypothesize, that ATO interferes with the HAdV characteristic reorganization of PML‐NBs into track‐like structures, as well as with efficient formation of viral replication centers, probably by interference with E2A SUMOylation, which ultimately results in a reconstitution of PML‐NBs and inhibition of HAdV replication.

Taken together, here we show that ATO blocks viral transcription and replication. We hypothesize a process by which reformation of PML nuclear bodies during HAdV infection would abrogate the beneficial effect of certain components of PML‐NBs (**Figure** **6**). These factors cannot be retained in E4orf3 induced track structures containing PML, and thus show no close vicinity to RCs where active viral transcription takes place. The failure in PML‐NB disruption also results in the inability of the viral E1B‐55K/E4orf6‐based E3‐ubiquitin ligase complex to access its substrates, and therefore also impairs efficient counteraction of antiviral mechanisms of the host cell. We additionally propose that the hyper‐SUMOylation of PML during ATO treatment induces a depletion of the host cellular SUMO pool. This deregulation of the SUMO PTM, which crucially regulates the HAdV life cycle, might contribute to the block in efficient HAdV replication induced by ATO treatment. Due to raising numbers of transplantation cases and thus more immunosuppressive treatments in the clinics, we actually note the increased incidence of severe HAdV infections. As adequate antiviral treatment options are not available, we cannot control severe HAdV infections and improve health conditions of immunosuppressed patients with HAdV infections. Here, we suggest ATO as a future treatment option, that blocks productive HAdV replication, targets host structures, and secures host antiviral measurements in the patient.

## 4 Experimental Section

##### Cell Culture and Cell Lines

H1299 (ATCC, No. CRL‐5803), HEK293 cells (ECACC European Collection of Authenticated Cell Cultures; Sigma Aldrich, No. 85120602‐1VL), and A549 cells (ATCC, No. CCL‐185) were grown in Dulbecco's modified Eagle's medium supplemented with 5% fetal calf serum, 100 U of penicillin, 100 µg of streptomycin per mL in a 5% CO_2_ atmosphere at 37 °C. All cell lines were frequently tested for mycoplasma contamination.

##### ATO Treatment

ATO was purchased from Sigma Aldrich (St. Louis, MO) and dissolved in 0.1 m NaOH to a stock concentration of 100 mm and diluted to 1 mm in PBS. For the treatment of infected cells, the inoculum was removed, and the medium was exchanged to medium containing ATO in the depicted concentrations.

##### Plasmids and Transient Transfection

p6xHis‐SUMO2 was kindly provided by Dr. Ron T. Hay (University of Dundee, UK). For transient transfections, subconfluent H1299 cells were transfected with a mixture of linear polyethylenimine (PEI; 25 kDa) and the respective DNA, as described before.^35^

##### Cytotoxicity and Cell Viability Assays

Cell viability in response to infection and treatment with ATO was determined 24–48 h p.i. treatment using the Promega (Madison, WI) CellTiter‐Blue Cell Viability Assay system according to the manufacturer's manual. Fluorescence values were detected using a Tecan (Männedorf, Switzerland) Infinite 200M plate reader.

##### Viruses

H5*pg*4100 served as the wild‐type (wt) virus.^105^ In addition, a replication competent HAdV‐C5 delta E3 virus, encoding a CMV promoter driven eGFP expression cassette, as well as an eGFP expressing HAdV‐C5‐based first generation adenoviral vector were generated and used in this study. All viruses were propagated and titrated in HEK293 cells. For this, infected cells were harvested after 48 h p.i. and lysed three times of freeze and thaw and reinfected into HEK293 cells. Virus growth was determined by immunofluorescence staining of the adenoviral DNA binding protein E2A.

##### Tecan Measurement

Subconfluent H1299 cells in 96‐well plates were infected with either wt HAdV‐C5, a replication competent HAdV‐C5 deltaE3 virus, encoding a CMV promoter driven eGFP expression cassette, or an eGFP expressing HAdV‐C5‐based first generation adenoviral vector. Two hours p.i., the inoculum was removed and the cells were treated with serial dilutions of ATO in the respective medium. For GFP expressing viruses, the GFP fluorescence was measured 24 h, as well as 48 h p.i. using a Tecan Infinite 200M plate reader using an excitation and emission wavelength of 488 and 520 nm. For HAdV‐C5 wt, E2A and capsid proteins were stained by indirect immunofluorescence and fluorescence intensity was measured using a Tecan Infinite 200M plate reader using an excitation and emission wavelength of 488 and 520 nm for Alexa 488 (E2A) and 640 and 670 nm for Alexa 647 (capsid), respectively.

##### Determination of Viral Infection Using the IncuCyte S3 Live‐Cell Analysis System

Subconfluent A549 cells in 96‐well plates were infected with a replication competent HAdV‐C5 delta E3 virus, encoding a CMV promoter driven eGFP expression cassette. Two hours p.i., the inoculum was removed and the cells were treated with serial dilutions of ATO in the respective medium. GFP expression and cell growth were monitored at four positions per well for 48 h with a 2 h increment using the Sartorius (Göttingen, Germany) IncuCyte S3 Live‐Cell Analysis System.

##### Antibodies and Protein Analysis

Primary antibodies specific for adenoviral proteins included E1B‐55K mouse mAb 2A6,^106^ E4orf6 mouse mAb RSA3,^107^ E4orf3 mouse mAb 6A‐11,^108^ E2A‐72K mouse mAb B6‐8,^109^ E1A mouse mAb M73,^110^ and HAdV‐5 rabbit polyclonal serum L133.^111^ Primary antibodies specific for cellular proteins included monoclonal mouse Ab against the 6xHis epitope (631 213; Clontech), polyclonal rabbit Ab raised against the PML protein (NB100‐59787; Novus Biologicals), Sp100 rabbit pAb GH3 (kindly provided by Prof. Hans Will), Mre11 rabbit pAB pNB 100‐142 (Novus Biologicals, Inc.), and ß‐actin mouse mAb AC‐15 (Sigma‐Aldrich, Inc.).

Secondary antibodies conjugated to horseradish peroxidase (HRP) for the detection of proteins by immunoblotting were anti‐rabbit IgG, anti‐mouse IgG, anti‐mouse light chain IgG, and anti‐rat IgG (Jackson/Dianova). All protein extracts were prepared in RIPA lysis buffer as described recently.^103^ To detect SUMO2 PTM of E2A, cells were transfected with p6His‐SUMO2 constructs, cell harvesting was performed at 4 °C, and denaturating purification was performed as described in ref. 43. Proteins were separated by SDS‐PAGE, transferred to nitrocellulose blotting membranes (0.45 µm) and visualized by immunoblotting. Autoradiograms were scanned and cropped using Adobe Photoshop CS5 and figures were prepared using Adobe Illustrator CS5 software. Fiji^112^ was used for quantitative analysis and amount comparison of western blots.

##### Indirect Immunofluorescence

For indirect immunofluorescence, H1299 cells were grown on glass coverslips in 1.5 × 10^5^ cells per well. At different times, cells were fixed in 4% paraformaldehyde (PFA) for 20 min at 4 °C or with ice‐cold ethanol for 10 min at −20 °C. Subsequently, cells were permeabilized in PBS with 0.5 Triton X‐100 for 5 min at room temperature. After 15 min blocking in Tris‐buffered saline‐BG (TBS‐BG; BG is 5% w/v BSA and 5% w/v glycine), buffer coverslips were treated for 30 min with the indicated primary antibody diluted in PBS, washed three times in TBS‐BG. After 20 min incubation with the corresponding Alexa 488 (Invitrogen)‐ or Alexa 647 (Dianova)‐conjugated secondary antibodies, they were washed two times in TBS‐BG and one time in PBS. The coverslips were then mounted in Glow medium (Energene) and digital images were acquired with a confocal laser‐scanning microscope (Nikon). Images were sampled and analyzed using Volocity software.

##### HAdV RNA and DNA Synthesis

Viral RNA was isolated from cells and reverse transcribed after protocols summarized in ref. 113. Quantitative RT‐PCR was performed in a LightCycler 480 (Roche) using 4 µL of 1/10 diluted cDNA, 10 pmol µL^−1^ of the corresponding oligonucleotide primers, and 5 µL of SYBR Green Mastermix (Roche) per sample. The following PCR conditions were used: 10 min at 95 °C and 40 cycles of 30 s at 95 °C, 30 s at 62 °C, and 30 s at 72 °C. The viral mRNA levels obtained from triplicate reactions were calculated in relation to levels of the cellular 18S mRNA. The following primers were used (forward and reverse primer): E1A (GTGCCCCATTAACCAGTTG, GGCGTTTACAGCTCAAGTCC), Hexon (CGCTGGACATGACTTTTGAG, GAACGGTGTGCGCAGGTA), 18S (CGGCTACCACATCCAAGGAA, GCTGGAATTACCGCGGCT). For the analysis of viral DNA, protein lysates were digested with Proteinase K (PK) for 1 h at 55 °C and boiled afterward for 10 min at 95 °C to inactivate the PK. Samples were then subjected to quantitative PCR analysis in a LightCycler 480 (Roche) using 4 µL of 1/200 diluted DNA, 10 pmol µL^−1^ of the corresponding oligonucleotide primers and 5 µL of SYBR Green Mastermix (Roche) per sample. The following PCR conditions were used: 10 min at 95 °C and 40 cycles of 30 s at 95 °C, 30 s at 62 °C, and 30 s at 72 °C. The viral DNA levels obtained from triplicate reactions were calculated in relation to levels of the cellular *gapdh* coding region. The following primers were used (forward and reverse primer): E1B‐55K (ATGAGCGACGAAGAAACCCATCTGAGC, CGGTGTCTGGTCATTAAGCT), GAPDH (CATCCTGGGCTACACTGA, TTGACAAAGTGGTCGGTTG).

##### Viral Capsid Formation

Cells for the determination of viral capsid formation were resuspended in a low stringent lysis buffer (50 mm Tris‐Cl (pH 8.0), 100 mm NaCl, 1 mm EDTA, and 1% NP‐40) and incubated for 10 min on ice. Samples were centrifuged at 12 000 × *g* at 4 °C. Samples were mixed with 6× loading buffer (50% glycerol and 0.1% bromophenol blue) and subjected to agarose gel electrophoresis. Proteins were transferred to nitrocellulose blotting membranes (0.2 µm) by capillary transfer using 10× saline sodium citrate (SSC) buffer and visualized by immunoblotting. Autoradiograms were scanned and cropped using Adobe Photoshop CS5 and figures were prepared using Adobe Illustrator CS5 software. Fiji^112^ was used for quantitative analysis and amount comparison of western blots.

##### Statistical Analyses

Testing for statistically significant differences in mean values was performed using a one‐way ANOVA and Dunnet's T3 test. For differences in proportions, a chi‐square test was performed. All statistical evaluations were performed using the GraphPad (San Diego, CA) Prism5 software.

## Conflict of Interest

The authors declare no conflict of interest.
